# Epidemiology of adult overweight recording and management by UK GPs: a systematic review

**DOI:** 10.3399/bjgp17X692309

**Published:** 2017-08-29

**Authors:** Joanna C McLaughlin, Kathryn Hamilton, Ruth Kipping

**Affiliations:** South West Public Health Training Programme;; South West Public Health Training Programme;; School of Social and Community Medicine, University of Bristol, Bristol.

**Keywords:** body mass index, general practice, obesity, primary health care, weight recording

## Abstract

**Background:**

Primary care guidelines for managing adult overweight/obesity recommend routine measurement of body mass index (BMI) and the offer of weight management interventions. Many studies state that this is rarely done, but the extent to which overweight/obesity is recognised, considered, and documented in routine care has not been determined.

**Aim:**

To identify the epidemiology of adult overweight documentation and management by UK GPs.

**Design and setting:**

A systematic review of studies since 2006 from eight electronic databases and grey literature.

**Method:**

Included studies measured the proportion of adult patients with documented BMI or weight loss intervention offers in routine primary care in the UK. A narrative synthesis reports the prevalence and pattern of the outcomes.

**Results:**

In total, 2845 articles were identified, and seven were included; four with UK-wide data and three with regional-level data. The proportion of patients with a documented BMI was 58–79% (28–37% within a year). For overweight/obese patients alone, 43–52% had a recent BMI record, and 15–42% had a documented intervention offer. BMI documentation was positively associated with older age, female sex, higher BMI, coexistent chronic disease, and higher deprivation.

**Conclusion:**

BMI is under-recorded and weight loss interventions are under-referred for primary care adult patients in the UK despite the obesity register in the Quality and Outcomes Framework (QOF). The review identified likely underserved groups such as younger males and otherwise healthy overweight/obese individuals to whom attention should now be directed. The proposed amendment to the obesity register QOF could prompt improvements but has not been adopted for 2017.

## INTRODUCTION

Most obese and overweight people in the UK state that they actively want to lose weight and would welcome advice from their doctor, but only 42% of obese adults report ever having received weight management advice from a healthcare professional.^1^^,^^2^ National Institute for Health and Care Excellence (NICE) guidelines recommend routine identification of obesity in primary care including the use of body mass index (BMI) as a practical estimate of adiposity in adults.^3^^,^^4^ Around three-quarters of patients see their GP at least once a year and obese individuals are proportionately higher users of care.^5^

There is evidence that doctors are inaccurate when asked to estimate the weight of patients^6^^–^10 or even when taking anthropometric measurements that should be objective.^11^ In a UK study requiring GPs to estimate patients’ BMI from photos, all underestimated and estimations worsened as patient BMI increased.^12^ Although lack of recognition may play a role in under-documentation of BMI, some studies report lower rates of documentation than rates of self-report of weight discussion or diagnosis in a given consultation.^13^^,^^14^ Lack of documentation does not necessarily mean lack of recognition, but may reflect lack of weight prioritisation or intent to offer management. The documentation of a diagnosis of overweight or obesity itself in the patient’s record is important as it is associated with the patient’s receipt of interventions and weight management.^15^^,^^16^ GP intervention for weight management has been shown to be effective and acceptable to patients even when it is extremely brief.^17^

The QOF (Quality and Outcomes Framework) is a voluntary incentive programme that financially rewards GP practices in England for provision of ‘quality care’ with points assigned for performance against various indicators. In the 2015–2016 QOF, all practices in England received the points available for the stated QOF of *‘establishing and maintaining a register of patients aged ≥18 years who have a recorded BMI of ≥30 within the previous 12 months’*.^18^ The registers indicated an adult obesity prevalence of 9.5%. In contrast, the most recent (2014) Health Survey for England survey showed a considerable discrepancy, with 24% of males and 27% of females aged >16 years being obese. Some of this difference can be accounted for by any patient not having been seen in the previous 12 months being omitted from the register, but as obese patients require more health services — 74% have a comorbidity and at least three-quarters of the whole population see their GP each year^5^^,^^19^ — it is likely that the difference cannot be accounted for by this alone. As it has not been determined how complete and accurate each obesity register is, the register data are limited in gauging GPs’ success in identifying and recording their patients’ BMI. NICE proposed an additional obesity QOF indicator: *‘The percentage of patients aged 18 or over … who have had a record of a BMI being calculated in the preceding 5 years (and after their 18^th^ birthday)’*, but this has not been adopted in 2017.^20^ QOF has an uncertain future; already abandoned by Scotland, there are suggestions that England may follow suit.^21^

**How this fits in**

Adult overweight/obesity documentation or management by UK GPs has not been objectively quantified. This systematic review shows that around half of overweight/obese patients had a recent body mass index (BMI) record. The proportion of patients with a documented offer of weight loss intervention varied widely from 15% to 42%. The proposed Quality and Outcomes Framework indicator of *‘patients aged 18 or over … who have had a record of a BMI being calculated in the preceding 5 years’* could prompt improvements.

Many qualitative studies have been published highlighting the numerous barriers and difficulties that GPs feel they face in tackling obesity in primary care, including raising the issue with patients.^22^^,^^23^ There is, however, no clear source of quantitative data on the decisions taken by GPs faced with an overweight/obese patient in routine appointments, nor for the proportion and type of overweight/obese patients who are identified and documented as such.

In recognition of this gap in knowledge, this systematic review aimed to identify, collate, report, and interpret the available quantitative data on documentation of adult overweight and obesity by GPs in the UK.

## METHOD

### Search strategy

A systematic search using a predefined search protocol (available from authors on request) was carried out in June 2016. The following databases were searched: MEDLINE, EMBASE, CINAHL Plus, ASSIA, HMIC, BNI, Cochrane Library, and the Index to Theses. Limits were placed on all searches to English-language articles and to exclude articles published before 2006, in recognition of the introduction of the obesity register QOF.

All retrieved studies were saved to RefWorks reference manager software. Duplicates were removed. One author screened the titles, abstracts, and full text of articles to determine inclusion. A second author screened a random subset of 10% of the articles at each stage with discussion over any discrepancies. An inter-rater reliability score was calculated using Cohen’s κ.

The secondary sources comprised hand searching of the reference lists and citations of key papers found in databases, and e-mail communication with key authors to request details of any further studies meeting the inclusion criteria.

### Inclusion criteria

To be eligible for inclusion, the studies had to report quantitative data on an objective measure of GPs’ documentation of routine recording/discussion/diagnosis of BMI/weight/lifestyle advice/offering of weight loss intervention as a main outcome. Studies had to be set in primary care in the UK and the subjects were adults (aged ≥16 years). Studies reporting on only a narrow group of patients (for example, patients with diabetes) or on a specialised setting (for example, a weight management clinic) were not eligible for inclusion.

### Critical appraisal and data extraction

A bespoke critical appraisal tool (Table 1) that incorporated all important aspects of study quality, irrespective of the mix of study designs to allow improved comparability between studies, was piloted and then applied to each paper by two authors individually. The quality assessment tool developed here is based on methodology used in other reviews,^24^^–^26 and the Critical Appraisal Skills Programme (CASP) recommended checklists for each different study type where available, in particular the Newcastle–Ottawa scale for cohort and case control studies.^27^ For cross-sectional studies the Joanna Briggs Institute tool for appraisal of analytical cross-sectional studies^28^ and questions adapted from Guyatt *et al*’s publications on descriptive and cross-sectional studies were used for guidance.^29^^,^^30^

**Table 1.** Summary of quality assessment tool results

	**Lead author**						

	**Artac^34^**	**Bhaskaran^35^**	**Booth^32^**	**Booth^31^**	**Dalton^36^**	**Goodfellow^37^**	**Osborn^33^**

Year of publication	2013	2013	2015	2013	2011	2016	2011

Rationale and aim clear?	×	×	×	×	×	×	×

Appropriate study design?	×	×	×	×	×	×	×

Baseline demographics of subjects given?	×		×	×	×	×	×

**Population choice, representative of:**							
UK primary care patients nationally		×					
UK primary care patients in a local area							
A specific group	×		×	×	×	×	×
Not specified							

Predefined sampling frame?	×	×	×	×	×	×	×

**Sampling type:**							
Census/100% sample	×				×		
Random		×	×	×		×	
Systematic							×
Convenience							
Not specified							

Setting and location of recruitment identified?	×	×	×	×	×	×	×

Applied equally to all subjects?	×	×	×	×	×		×

Validated/standardised extraction technique?	×				×	×	

Data from quality-controlled database of secure records?	×	×	×	×	×	×	×

Time period included clear (post-QOF data identified)?	×	×	×	×	×	×	×

Clearly defined outcome measures, for example, BMI-defined obesity	×	×	×	×	×	×	

Primary outcome was BMI or calculable BMI record	×	×		×	×		×

Medical codes/other documentation included			×	×	×	×	

Predictive factors, for example, age, sex, practice detailed	×	×	×	×	×	×	×

Statistical methods used appropriately	×	×	×	×	×	×	×

Results presented clearly (sufficient data presented)	×	×	×	×	×		

Interpretation takes into account sources of bias/imprecision (exclusions, missing data)	×	×	×	×	×	×	×

Interpretation is made in the context of current evidence	×	×	×	×	×	×	×

**Generalisability of results:**							
All UK adults							
All UK primary care adult patients		×					×
All overweight/obese UK primary care patients			×	×		×	
Patients in a local region	×				×		
Not clear to whom generalisable							

*BMI = body mass index. QOF = Quality and Outcomes Framwork.* × *= yes.*

A narrative synthesis was prepared for the papers that met the exclusion and inclusion criteria. Emphasis was placed on interpreting and presenting the heterogeneity between studies and the individual risk of bias present for each outcome measure.

## RESULTS

**Figure 1.**
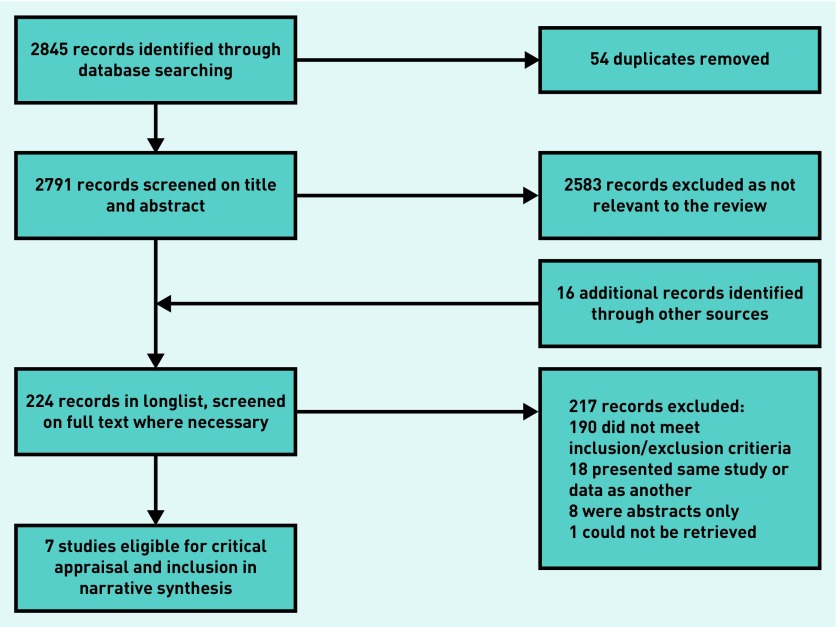
***Flowchart of selection process.***

The search strategy returned 2845 results through searches of the electronic databases, 208 of which were retained after screening the titles and abstracts for relevance to the review and removal of duplicate results (Figure 1). A further 16 results from additional sources, from hand searches of reference lists and citations of key papers, were identified and subjected to further screening for suitability. Responses were received from four of the key authors, whose guidance had been requested by e-mail on any further studies suitable for inclusion in the review. No further studies were identified. Full-text versions of the papers were retrieved at this stage. Seven studies met the inclusion and exclusion criteria.

The Cohen’s κ was 1.00 for agreement between the two authors at both the title screening and full-text stages.

Of the seven studies that were included in the review (Table 2): three^31^^–^33 were retrospective cohort studies based on large primary care databases, three^34^^–^36 were cross-sectional/descriptive in design using data from a primary care database or regional GP practice patient records, and the final study^37^ was a cluster randomised controlled trial within a further region of GP practices. The quality assessment tool allows visual comparison of the areas of difference and similarity in quality across the papers (Table 2). Table 3 presents the results of the outcome measures for each study.

**Table 2.** Studies meeting inclusion and exclusion criteria^a^

**Study**	**Primary care subjects^b^**	**Study’s design and relevance**
**Artac, 2013^34^** *Evaluation of a National Cardiovascular* *Risk Assessment Programme (NHS Health Check)* PhD thesis	Hammersmith and Fulham (London, UK) GP practices *N*= 42 306	Cross-sectional study of electronic medical records of patients eligible for NHS Health Checks, describes BMI recording completeness
**Bhaskaran *et al*, 2013^35^** Representativeness and optimal use of body mass index (BMI) in the UK Clinical Practice Research Datalink (CPRD)	UK-wide database; Clinical Practice Research Datalink (CPRD) *N*= 325 948	Descriptive study of completeness of BMI recording for a sample of >16-year-olds within this primary care database
**Booth *et al*, 2015^32^** Access to weight reduction interventions for overweight and obese patients in UK primary care: population-based cohort study	UK-wide database; Clinical Practice Research Datalink (CPRD) *N*= 91 413	Retrospective cohort study of recorded weight management intervention offers for a sample of overweight/obese patients within this primary care database
**Booth *et al*, 2013^31^** Epidemiology of clinical body mass index recording in an obese population in primary care: a cohort study	UK-wide database; General Practice Research Database (GPRD) *N*= 40 000–46 000 per year	Retrospective cohort study of the epidemiology of recording of BMI for a sample of obese patients within this primary care database
**Dalton *et al*, 2011^36^** Implementation of the NHS Health Checks programme: baseline assessment of risk factor recording in an urban culturally diverse setting	North West London (UK) GP practices participating in pilot NHS Health Checks Programme *N*= 21 510	Cross-sectional study of electronic medical records of patients eligible for NHS Health Checks, describes BMI recording completeness
**Goodfellow *et al*, 2016^37^** Cluster randomised trial of a tailored intervention to improve the management of overweight and obesity in primary care in England	East Midlands (UK) GP practices *N*= 32 079	Cluster randomised controlled trial of an intervention to improve obesity management in primary care. Data from the control arm describe BMI recording and interventions offered to overweight/obese patients aged >16 years in normal practice
**Osborn *et al*, 2011^33^** Inequalities in the provision of cardiovascular screening to people with severe mental illnesses in primary care. Cohort study in the United Kingdom THIN Primary Care Database 2000–2007	UK-wide database; The Health Improvement Network (THIN) *N*= 95 512	Retrospective cohort study describing BMI recording in patients aged >18 years within the database with serious mental illness and for controls (the group of interest here)

a *Where subjects are described as overweight or obese this refers to a BMI of* ≥*25 and* ≥*30 kg/m^2^, respectively.*

b N *refers to the number of subjects in the study relevant to the review research question. BMI = body mass index.*

**Table 3.** Summary of patient characteristics by study

**Study**	**Cohort**	**Source**	**Included patients, *n*^a^**	**GP practices, *n***	**Mean age, years**	**Male, %**	**Ethnic group**	**Socioeconomic status**	**Mean BMI**	**Proportion with BMI record (within specified timeframe)^b^**	**Proportion with record of weight loss intervention**
Artac 2013^34^	40–74 years without known CVD or diabetes	Hammersmith and Fulham GP practices	42 306	28	52.2	46.2	35.9% white British,	‘Diverse’ 2.5% South Asian, and 8.6% black (data missing 22.3%)	26.6	Males: 57.9% Females: 60.7% (within 5 years) in 2008/2009	Not measured
Bhaskaran 2013^35^	>16 years with any historical BMI record	CPRD database	325 948	–	-–	–	–	–	∼26	79% (any record) 52% (within 3 years) in 2011	Not measured
Dalton 2011^36^	35–74 years without known CVD or diabetes	North West London GP practices	21 510	14	50.2	52.9	38.5% white, 39.6% South Asian, 10.4% black (data missing for 55%)	More deprived than the UK	– Females: 67.6% (within 5 years) in 2008/2009	Males: 79.3%	Not measured
Osborn 2011^33^	>18 years without SMI or CVD	THIN database	95 512	420	53.2	47.4	–	Similar to the UK	–	Males: 27.6% Females: 37.3% (within 1 year) in 2007	Not measured
Booth 2015^32^	>18 years, BMI ≥25 record within the study period	CPRD database	91 413	491	56	53	–	Low levels of deprivation	–	All, by definition	Morbidly obese: males 40%, females 41.9% Non-morbidly obese: males 15.8%, females 19.8% within 2005–2012
Booth 2013^31^	>18 years, BMI ≥30 or medical code for obesity 1997–2007	GPRD database	40 728	127	–	41	–	Higher numbers in most deprived quintiles	37 female 35.5 male	Males: 45.6% Females: 52.1%, within 2009	Not measured
Goodfellow 2016^37^	>16 years, BMI ≥25 before the study period	East Midlands GP practices	32 079	16	50	47.5	65.6% white, 16.7% South Asian, 6.3% black	More deprived than the UK	30.2 (SD 5.4) average	42.7% (SD 10.3%)^c^ within 11-month study period 2013/2014	15.1% (SD 10.8%) offered within 11-month study period 2013/2014

a Only from the group and time period of meeting inclusion criteria. The study may have reported on a larger group overall.

b Data shown are from most recent time period if the study reported on multiple periods.

c *BMI or waist circumference recorded. – = not stated. Morbidly obese refers to a BMI* ≥*40 kg/m^2^. BMI = body mass index. CVD = cardiovascular disease. CPRD = Clinical Practice Research Datalink. GPRD = General Practice Research Database. SD = standard deviation. SMI = serious mental illness. THIN = The Health Improvement Network.*

### Proportion of patients with a documented BMI

The proportion of adult patients with any record of BMI was 79%; however, when the recording period was recent, this decreased to between 57.9% and 79.3% for males and 60.7% and 67.6% for females when considering the previous 5 years,^34^^,^^36^ and to 52% for the previous 3 years.^35^ Further, when BMI documentation was considered within 11–12 months, which is closer to the QOF register, this ranged from 27.6% to 45.6% for males and 37.3% to 52.1% for females.^31^^,^^33^^,^^37^

### Proportion of patients with a documented offer of a weight management intervention

The offer of a weight management intervention was measured in two studies: 15.1% of overweight or obese adults were offered an intervention in one 11-month period studied;^37^ in another study the proportions varied greatly by level of overweight and obesity, with approximately 40% and 41.9%, respectively, of morbidly obese males and females being offered an intervention, while this reduced to 15.8% and 19.8% of non-morbidly obese males and females, and 10% of overweight patients.^32^

### Patient factors associated with the outcomes

All the studies described a pattern of association between BMI recording and increasing patient age, and also with female sex. Several studies showed higher rates of BMI recording for older patients, higher BMI, or comorbidities, and suggested that this was because these groups tend to consult primary care practitioners more often. Five studies^31^^–^34^,^^36^ reported that BMI recording or weight loss intervention offer rates were associated with increasing deprivation at the patient level (generally based on postcode).

## DISCUSSION

### Summary

This systematic review included seven studies in total: four large UK-wide studies and three regional studies with either a BMI record or an offer of weight loss intervention documented for adult patients in routine primary care consultations in the UK. The general proportion of adult patients with a documented BMI was reported at 58–79% over the longer term, and 28–37% where the record was within 12 months. The proportion of overweight/obese patients with a recent documented BMI record was 43–52%. The proportion of overweight/obese adult patients with a documented offer of weight loss intervention was reported in a less consistent manner and ranged from 15% to 42%. Regional settings of the data collection and different timeframes for the intervention offer may account for the wide variation.

Higher rates of documentation were associated with patients’ older age, female sex, higher BMI, coexistent chronic disease, and higher deprivation. This was attributed to primary care consultation rates being higher for these groups,^38^^,^^39^ leading to increased opportunity for recognition and recording.

### Strengths and limitations

The comprehensive nature of the search strategy, with the inclusion of all study designs, is a strength of the study. Further, the collection of data from patient records allowed comparability among studies. Patient electronic health records, particularly within quality-controlled research databases, are a strong source of data for primary care research but they do not cover verbal exchanges or elements such as patient refusal.

Although the review included studies with adult patients served by the UK primary care system in the last 10 years, the external validity for some studies is limited by those that were regional databases or where studies only included overweight/obese patients and differing lengths of time for which documentation in the patient record was measured. A limitation of the review is that the classification of an ‘up-to-date’ BMI record varied among studies. Although the 2006 QOF indicator implies that the period of interest is 12 months, there is no consensus in the literature over how recent a BMI record needs to be to be representative. To deal with this variability, this review has reported on categories of BMI record recency. A further limitation is that it is possible that participants in longitudinal studies were not representative of the general primary care adult population, and awareness of being studied could have altered the behaviour of the primary care team or patients involved in the control arms.

The systematic review aimed to draw inferences for the whole UK adult primary care patient population, but it is challenging to account for adults who either are not registered with a GP or who choose not to consult their GP. Estimating the number of unregistered adults in the UK is not straightforward.^40^

### Comparison with existing literature

The results from the synthesis of the included papers in this review show coherence with the results of publications that have presented data before introduction of the QOF in 2006. In 2003, 42% of obese adults had a BMI recorded and 40% had been given weight management advice in a primary care study.^41^ A cross-sectional study from 2004 on the electronic GP records of 435 102 patients in England showed that 56.8% of males and 69.3% of females had a BMI record, supporting the finding of this review that females have higher rates of recording.^42^

This review did not include data from more subjective outcome measures such as patient self-report of weight loss discussion, but a study of this nature found that less than one-third of overweight or obese patients reported that they had received lifestyle advice for weight management from their GP,^43^ a figure within the range of the proportion of patients with a documented weight loss intervention offer in this review. A small study of 42 videotaped routine consultations by GPs in Scotland showed that weight management was rarely mentioned, even when the patient was overweight or obese.^44^

### Implications for research and practice

The growing burden of obesity on primary care and the discrepancies between data sourced in primary care and accepted measures of ‘true prevalence’ of overweight/obesity in the UK support the case for changes to policy and practice in this area.

A large, UK-wide observational study to gather contemporary data from routine consultations between adult patients and all members of the primary care team would be invaluable in considering the uncertainties surrounding undocumented recognition, discussion, and intervention for overweight/obesity, including an exploration of the role of patient refusal to be weighed or engage in weight management.

Future studies would benefit from closer integration with the policy context, for example, consistent use of outcome measures that reflect the values and recommendations of the NICE guidelines and QOF indicators.

Structured recording of patient BMI and interventions offered could improve the overall prevalence of recognition of overweight/obesity and decrease the inequalities that likely result from the differences in practice between patient groups. The proposed QOF indicator addition — *‘The percentage of patients aged 18 or over … who have had a record of a BMI being calculated in the preceding 5 years (and after their 18^th^ birthday)’* — may prompt improvements, although the obesity register QOF has remained unchanged for 2017 and the future of QOF in England is uncertain generally. Complementary interventions may include electronic prompts to record patients’ weight at registration or yearly intervals and facilities for patients to submit their own weight record remotely. Attention should be paid to the patient groups revealed by this review to be most underserved, such as younger males and overweight/obese individuals with no comorbidities.

Qualitative research suggests that some GPs believe patients carry the responsibility for their obesity and that primary care is not the appropriate source of intervention,^45^^,^^46^ and GP motivation to consider weight is damaged by a real and perceived lack of available and effective interventions.^5^^,^^32^^,^^47^ This presents a challenge to the improvement of weight management’s integration into a primary care system already struggling with capacity. In contrast, public health and obesity experts view obesity as a chronic disease, and maintain that primary care healthcare professionals are vital in dealing with the problem.^48^
